# Hyperbaric oxygen in chronic traumatic brain injury: oxygen, pressure, and gene therapy

**DOI:** 10.1186/s13618-015-0030-6

**Published:** 2015-07-14

**Authors:** Paul G. Harch

**Affiliations:** Section of Emergency Medicine, Department of Medicine, Louisiana State University School of Medicine, 1542 Tulane Avenue, Rm. 452, Box T4M2, New Orleans, LA 70112 USA

**Keywords:** Hyperbaric, Oxygen, Traumatic, Brain, Injury, Concussion, Pressure, Gene, Therapy, Veteran

## Abstract

Hyperbaric oxygen therapy is a treatment for wounds in any location and of any duration that has been misunderstood for 353 years. Since 2008 it has been applied to the persistent post-concussion syndrome of mild traumatic brain injury by civilian and later military researchers with apparent conflicting results. The civilian studies are positive and the military-funded studies are a mixture of misinterpreted positive data, indeterminate data, and negative data. This has confused the medical, academic, and lay communities. The source of the confusion is a fundamental misunderstanding of the definition, principles, and mechanisms of action of hyperbaric oxygen therapy. This article argues that the traditional definition of hyperbaric oxygen therapy is arbitrary. The article establishes a scientific definition of hyperbaric oxygen therapy as a wound-healing therapy of combined increased atmospheric pressure and pressure of oxygen over ambient atmospheric pressure and pressure of oxygen whose main mechanisms of action are gene-mediated. Hyperbaric oxygen therapy exerts its wound-healing effects by expression and suppression of thousands of genes. The dominant gene actions are upregulation of trophic and anti-inflammatory genes and down-regulation of pro-inflammatory and apoptotic genes. The combination of genes affected depends on the different combinations of total pressure and pressure of oxygen. Understanding that hyperbaric oxygen therapy is a pressure and oxygen dose-dependent gene therapy allows for reconciliation of the conflicting TBI study results as outcomes of different doses of pressure and oxygen.

## Background

Confusion over the conflicting conclusions of recent civilian and United States Department of Defense (DoD) trials of hyperbaric oxygen therapy (HBOT) in the treatment of mild traumatic brain injury (mTBI) persistent post-concussion syndrome (PPCS) [1–6] have focused attention on critical flaws [7, 8] in the historical definition of HBOT [9] that beg the question “What is hyperbaric oxygen therapy?” The answer to this question has led to a re-appraisal of HBOT as a dual-component [7, 8] gene therapy [7] that is poised to not only change, but also expand the field of hyperbaric therapy.

## Main text

The historical definition of HBOT (“…a treatment in which a patient breathes 100 % oxygen …at… > 1 atmosphere absolute…pressurization should be to 1.4 atm abs or higher.”) [9] focuses solely on the absolute pressure of 100 % oxygen above 1.40 ATA. The 1.4 ATA threshold is both arbitrary and limiting when considering that, by definition, oxygen at 1.399999 ATA would not be hyperbaric oxygen therapy. Yet, there is no published data on a difference in clinical efficacy between 1.40 and 1.399999 ATA oxygen for any diagnosis. Furthermore, any 100 % oxygen exposure greater than ambient atmospheric pressure or between 1.0 and 1.4 ATA, or total pressurization between 1.0 and 1.4 ATA of oxygen-enriched breathing gas > .21 ATA oxygen would not be hyperbaric oxygen therapy. This excludes a substantial body of clinical literature [10], especially Russian hyperbaric literature where pressures between 1.1 and 1.4 ATA were common (See abstracts of 7th International Congress on Hyperbaric Medicine, Moscow, 1981). The definition is also limiting by its exclusion of the acknowledged bioactivity of pressure [7, 11]. While relevant in studies where beneficial effects in pressurized air control groups have been attributed to the increased partial pressure of oxygen [12, 13], it is also relevant to the erroneous claim in DoD studies [3–5] that the 2.0 ATA/normoxic control group is a sham. Both the sham claim and the historical definition of HBOT are further erroneous when considering that every clinical HBOT is a combination of increasing partial pressure of oxygen and total pressure during pressurization and for at least the first 18 min of treatment [14], and decreasing partial pressure of oxygen during decompression.

More accurately, the definition of HBOT is a therapy of increased total atmospheric pressure and partial pressure of oxygen over ambient total and oxygen partial pressures [7, 8]. The bioactivity of increased 100 % total atmospheric pressure oxygen is well known [7–9]. The bioactivity of increased atmospheric pressure is unknown to the clinical hyperbaric medicine community, but well-documented in an extensive basic science literature [11]. Dozens of investigators have reported widespread biological effects of increased pressure across the entire phylogenetic spectrum that begin as early as 30 s after compression [11].

HBOT In the United States is primarily applied to acute and chronic wound conditions and certain infections [9]. Infections are wound conditions due to the effects of the inflammatory reaction and scar formation. HBOT has a wide range of effects on wound pathophysiology [9]. The daily input of HBOT produces wound healing. HBOT heals wounds by the trophic processes of blood vessel, connective tissue, bone, and skin growth [15–18]. These trophic effects require mitotic activity, however, the intermediary steps for trophism were a void in hyperbaric science until 1997 [19].

Siddiqui, et al. [19] proposed that HBOT was a deoxyribonucleic acid (DNA) signaling agent based on wound-healing synergy of oxygen and growth factors and an HBOT-induced change in the oxygen capacitance of ischemic animal wounds. Multiple studies on HBOT-generated single gene products followed [20–32]. Recently, gene array analyses have demonstrated widespread gene expression/suppression effects of hyperoxia and/or increased atmospheric pressure: **1**) Cells grown in 2 ATA air (~.40 ATA oxygen) versus cells in 40 % oxygen at 1 ATA expressed cell adhesion, stress response, transcription, apoptosis, tumor suppressor-related, and mitogen-activated protein kinase-related genes [33], **2**) Independent and overlapping genes are sensitive to increases in pressure, oxygen, or both [34], **3**) As many as 8101 genes were either up- or down-regulated over 24 h after a single exposure to HBOT [35] (upregulated genes were primarily growth and repair hormone and the anti-inflammatory genes; downregulated genes were the pro-inflammatory and apoptotic genes), and **4**) Differential suppression of inflammatory genes at 1.0, 1.5, and 2.4 ATA oxygen with maximal suppression at 1.5 ATA [36]. While the oxygen studies’ results are partially qualified by in vitro:in vivo oxygen partial pressure differences [34, 37], the pressure results are not. The unqualified conclusion is that a substantial number of human genes are sensitive to increased atmospheric pressure, hyperoxia at increased atmospheric pressure, or both.

The lack of appreciation of the dual-component nature of hyperbaric oxygen therapy and the bioactivity of both pressure and hyperoxia at increased atmospheric pressure is widespread, but most evident in the recent DoD trials of HBOT in mTBI PPCS [1–6]. A review in Medical Gas Research [38] correctly mentions that one of the DoD studies does “…not address any potential therapeutic benefit of higher pressures in the absence of increased oxygen tension,” however, it does not elaborate on the literature describing bioactivity of pressure. An earlier review [39] mentions only the oxygen component of HBOT. A third review [40] noted, “Unfortunately, agreement that HBOT has a positive effect on TBI has not yet been reached due to the difference in external conditions.” Absent in this review was a discussion on the different doses of HBOT used in the various studies and the erroneous assumption in two of the studies that the “sham” groups were not treatment groups that used different doses of hyperbaric therapy. This erroneous assumption is present in all of the DoD mTBI HBOT PPCS studies [7, 8]. When viewed as multi-dose studies the results of the DoD studies become congruent with the results of civilian studies [41–44], suggesting effectiveness of some doses of hyperbaric therapy [1, 6, 41–44], ineffectiveness of others [3–5], and harm of another [2]. This appreciation of dosing differences raises the question of potential effectiveness of many other doses of pressure and hyperoxia in mTBI PPCS. They also spawn a rethinking and re-appraisal of the disputed historical claims of efficacy of HBOT in the treatment of well over one hundred diseases [45] dating to 1662, and the widely differing number of treatable indications in less scientifically restrictive countries, e.g. China [46], versus the United States.

## Conclusions

In conclusion, HBOT is the use of increased total atmospheric pressure and partial pressure of oxygen over ambient total and oxygen partial pressures to treat various disease processes and their diseases. The combination of increased atmospheric pressure and hyperoxia express or suppress upto 8101 genes in human cells [35]. Hyperbaric oxygen therapy appears to be the oldest, most enduring, and most effective gene therapy. Physicians and researchers are playing a symphony with gene expression and suppression, the combination of which is dependent on the different total pressures and partial pressures of oxygen. It is apparent that dosing of hyperbaric therapy is in its infancy, particularly in the pressure ranges from 1–2 ATA and across the spectrum of unexplored fractional inspired oxygen concentrations at pressures ≥ 1 ATA. It is also apparent that multiple doses of hyperbaric therapy are effective in the treatment of PPCS while others are not. With an appreciation of the scientific definition of hyperbaric oxygen therapy the field of Undersea and Hyperbaric Medicine is poised to rapidly expand with investigation of the lower dosing ranges of pressure and hyperoxia for a multitude of diagnoses.

## Abbreviations

ATA: Atmospheres absolute
DNA: DeoxyriboNucleic acid
DoD: United States department of defense
HBOT: Hyperbaric oxygen therapy
mTBI: Mild traumatic brain injury
PPCS: Persistent post-concussion syndrome
